# Perivascular adipose tissue promotes vascular dysfunction in murine lupus

**DOI:** 10.3389/fimmu.2023.1095034

**Published:** 2023-03-16

**Authors:** Hong Shi, Brandee Goo, David Kim, Taylor C. Kress, Mourad Ogbi, James Mintz, Hanping Wu, Eric J. Belin de Chantemèle, David Stepp, Xiaochun Long, Avirup Guha, Richard Lee, Laura Carbone, Brian H. Annex, David Y. Hui, Ha Won Kim, Neal L. Weintraub

**Affiliations:** ^1^ Division of Rheumatology, Medical College of Georgia, Augusta University, Augusta, GA, United States; ^2^ Vascular Biology Center, Medical College of Georgia, Augusta University, Augusta, GA, United States; ^3^ Department of Radiology and Imaging, Medical College of Georgia, Augusta University, Augusta, GA, United States; ^4^ Division of Cardiology, Department of Medicine, Medical College of Georgia, Augusta University, Augusta, GA, United States; ^5^ Department of Physiology, Medical College of Georgia, Augusta University, Augusta, GA, United States; ^6^ Department of Surgery, Medical College of Georgia, Augusta University, Augusta, GA, United States; ^7^ Department of Pathology and Laboratory Medicine, University of Cincinnati, Cincinnati, OH, United States

**Keywords:** systemic lupus erythematosus, cardiovascular disease, perivascular adipose tissue, inflammation, vasorelaxation

## Abstract

**Introduction:**

Patients with systemic lupus erythematosus (SLE) are at elevated risk for Q10 cardiovascular disease (CVD) due to accelerated atherosclerosis. Compared to heathy control subjects, lupus patients have higher volumes and densities of thoracic aortic perivascular adipose tissue (PVAT), which independently associates with vascular calcification, a marker of subclinical atherosclerosis. However, the biological and functional role of PVAT in SLE has not been directly investigated.

**Methods:**

Using mouse models of lupus, we studied the phenotype and function of PVAT, and the mechanisms linking PVAT and vascular dysfunction in lupus disease.

**Results and discussion:**

Lupus mice were hypermetabolic and exhibited partial lipodystrophy, with sparing of thoracic aortic PVAT. Using wire myography, we found that mice with active lupus exhibited impaired endothelium-dependent relaxation of thoracic aorta, which was further exacerbated in the presence of thoracic aortic PVAT. Interestingly, PVAT from lupus mice exhibited phenotypic switching, as evidenced by “whitening” and hypertrophy of perivascular adipocytes along with immune cell infiltration, in association with adventitial hyperplasia. In addition, expression of UCP1, a brown/beige adipose marker, was dramatically decreased, while CD45-positive leukocyte infiltration was increased, in PVAT from lupus mice. Furthermore, PVAT from lupus mice exhibited a marked decrease in adipogenic gene expression, concomitant with increased pro-inflammatory adipocytokine and leukocyte marker expression. Taken together, these results suggest that dysfunctional, inflamed PVAT may contribute to vascular disease in lupus.

## Introduction

Systemic lupus erythematosus (SLE) is a heterogeneous systemic inflammatory autoimmune disorder that primarily affects women of childbearing age, characterized by profound dysregulation of immune responses and multiorgan involvement with high morbidity and mortality compared to the general population (1–3). Despite the reduction in SLE-associated mortality over the last several decades due to improvements in diagnosis and therapy, mortality due to cardiovascular disease (CVD) remains strikingly elevated (4). This is especially the case for young females with SLE, where the CVD risk can be up to 50-fold higher than aged-matched controls (5, 6). The CVD disease in patients with SLE is mainly characterized by premature and accelerated atherosclerosis (7). In addition, outcomes after coronary artery stenting are worse in patients with SLE (8), suggesting an enhanced response to vascular injury. While traditional Framingham risk factors (such as hypertension, hyperlipidemia, diabetes, and smoking) likely contribute to CVD in SLE, they cannot fully account for the increased risk. Thus, the pathogenesis of premature CVD in SLE may rely on factors unique to SLE itself (7, 9, 10). While several theories have been proposed (11, 12), the underlying mechanisms have not been defined, nor have effective treatment strategies been developed.

Although prior studies suggest that adipose tissues are a source of chronic inflammation that may play an active role in atherosclerosis in SLE (13–16), the specific adipose tissue depots contributing to vascular disease in SLE have not been identified. Perivascular adipose tissue (PVAT) is a unique adipose tissue depot which surrounds most vessels except the cerebral vasculature. While it was initially thought to simply provide structural support to blood vessels, PVAT is now recognized to possess distinct endocrine/paracrine functions that regulate vascular homeostasis. In healthy states, PVAT may resemble thermogenic brown or beige adipose tissue and play a protective role in vascular metabolism and function. Conversely, in the setting of high fat diet and other cardiovascular risk factors, PVAT becomes dysfunctional, exhibiting a white-like phenotype, associated with loss of thermogenic capacity, enhanced oxidative stress, and increased immune cell infiltration and expression of inflammatory cytokines/adipokines, thus promoting endothelial dysfunction and atherosclerosis (17–21).

Notably, computed tomography (CT) scanning in women with SLE has demonstrated higher volume and density (a marker of inflammation) of PVAT surrounding the thoracic aorta compared to heathy control subjects (22, 23). In addition, PVAT density was strongly associated with aortic calcification score in SLE patients independent of age, circulating inflammatory markers, CVD risk factors and body mass index (BMI) (23). Moreover, SLE patients demonstrated increased thoracic aortic adventitial thickness, which was associated with aortic atherosclerosis, abnormal stiffness, and eccentric vessel remodeling (24). These findings suggest that PVAT may be dysfunctional in patients with SLE, thus contributing to adventitial remodeling and CVD. However, virtually nothing is known regarding the biology or function of PVAT, or how dysfunctional PVAT might contribute to vascular disease, in SLE.

Using mouse lupus models (NZBWF1/J and MRL/lpr lines), which are prone to developing endothelial and vascular dysfunction compared to control mice (12, 25–27), we investigated the phenotype and function of PVAT in the context of active lupus.

## Materials and methods

### Animals

Breeding pairs of two lupus-prone mouse lines, lupus MRL/lpr (#000485) and control MRL/MpJ (#000486), and lupus NZBWF1/J (#100008) and control NZW/LacJ (#001058), were purchased from The Jackson Laboratory. Mice were bred and maintained in specific pathogen-free conditions in the animal facilities. They were housed in a controlled environment at 20-22^o^C with a 12 hr light/12 hr dark cycle. Food and water were provided *ad libitum* to all animals. Only female mice were used in the experiments described here. MRL and MRL/lpr mice were harvested at age of 7 weeks (baseline, before production of autoantibodies and proteinuria) and 14 weeks (active disease with proteinuria). NZW/LacJ and NZBWF1/J mice were harvested between 36 to 40 weeks of age (active disease with proteinuria). All animal care and experimental protocols complied with the National Institutes of Health Guide for the Care and Use of Laboratory Animals and relevant ethical regulations, and were approved by the institutional Animal Care & Use Committee of Augusta University (AU).

### Measurements of body composition, energy homeostasis, body temperature and blood pressure

Body composition of fat and lean mass was measured by nuclear magnetic resonance (NMR) spectroscopy (Bruker Minispec LF90II, Bruker, Billerica, USA) as previously reported and normalized to total body weight (28). The volume of oxygen consumption (VO_2_), carbon dioxide production (VCO_2_), spontaneous motor activity and food intake were measured using the Comprehensive Laboratory Monitoring System (CLAMS) (Columbus Instruments, Columbus, USA) (28). The respiratory exchange ratio (RER) was calculated from the ratio of VCO_2_ to VO_2_. Mice were individually placed into the sealed chambers with free access to food and water. The study was carried out in a room set at 22^o^C with 12-12 hr (6:00 am ~ 6:00 pm) light-dark cycles, and the measurements were carried out continuously for 72 hours after a 24 h acclimatization period. The data were averaged over 72 hr. Rectal body temperatures were measured by a BAT-12 thermometer (Physitemp, USA). Blood pressure was recorded in conscious, restrained mice at age of 7 weeks and 14 weeks using the CODA tail cuff blood pressure monitoring system (Kent Scientific, USA).

### Biochemical analyses

At age of 14 weeks, the mice were fasted for 5 hours and blood collected from the inferior vena cava. The fasting whole blood glucose was measured using an Alpha TRAK glucometer. The fasting plasma insulin, leptin and resistin levels were assessed by Luminex Assay using MILIIPLEX Metabolic Hormones Expanded Panel (Cat. # MMHE-44K-07, Millipore, Burlington, USA). Plasma adiponectin was assessed by Luminex Bio-Plex Pro Mouse Diabetes Adiponectin Assay (Cat. # 171F7002M, Biorad, Hercules, USA), according to manufacturer’s instructions. For detection of adiponectin, plasma samples were diluted 6400 times. The chemocytokine levels were measured by Flow Based Bead Array using mouse inflammation panel (Cat. # 740446, BioLegend, USA). Levels of the mast cell-specific protease MCPT-6 in the plasma were measured using the Mouse Mast Cell Protease-6/Mcpt6 ELISA Kit according to the manufacturer’s instructions (Thermofisher, USA). The plasma total cholesterol and triglyceride levels were measured with LabAssay^TM^ Cholesterol and LabAssay^TM^ Triglyceride Kits (FujiFilm Healthcare, Lexington, MA, USA) as previously described (29). Plasma lipoprotein profiles were determined as described previously (29). Briefly, pooled plasma from 4 MRL/lpr mice and 5 MRL control mice were subjected to FPLC gel filtration on two Superose 6 columns connected in series. For assessment of the production of autoantibodies in lupus mice, serum anti-double stranded DNA (anti-dsDNA) antibodies were quantified by ELISA according to the manufacturer’s instruction (Alpha Diagnostics). Mice were screened for proteinuria weekly with Uristix-4 (Siemens), and elevations confirmed by measuring urinary albumin and creatinine concentrations using a mouse albumin enzyme-linked immunosorbent assay (ELISA; Bethyl Laboratories) and a creatinine Assay Kit (BioAssay Systems) following the manufacturer’s protocols. Urinary albumin-to-creatinine ratios (UACR) were then calculated.

### Glucose tolerance test

Mice were fasted for 5 hr followed by intraperitoneal injection of glucose at 1g/kg body weight. Glucose levels were measured *via* tail vein by an Alpha TRAK glucometer at baseline and every 15 min up to 2 hr following glucose injection as previously described (28).

### Assessment of vascular function

Thoracic aortas were isolated and dissected into rings of 2-3 mm in length, with PVAT either removed or left intact. Isometric tension was recorded using a wire myograph system 620 (Danish Myo Technology A.S, Denmark). The rings with or without PVAT were equilibrated for 60 min and contracted two times with 120 mM KCl. Concentration-response curves were obtained after contracting vessels with phenylephrine (PE; 10^-6^ M), followed by acetylcholine (Ach; 10^-7^ M) to test endothelial integrity. The rings with or without PVAT were submaximally pre-contracted with PE at EC75 concentration and allowed to reach a stable tension. To examine endothelium-dependent relaxation, Ach (10^−9^ M to 10^−4^ M) was added cumulatively to the bath and a curve was generated. Finally, endothelium-independent relaxation (vascular smooth muscle response) was assessed by washing out PE and Ach and then repeating the experiment with PE contraction and cumulative addition of sodium nitroprusside (SNP) (10^−10^ M to 10^−5^ M) to the bath. Ach and SNP relaxation were expressed as percentage of PE contraction.

### Tissue harvesting

At age of 14 weeks, retroperitoneal (posterior to the kidneys), subcutaneous, gonadal, retroperitoneal, and brown adipose tissues, as well as mesenteric and thoracic perivascular adipose tissues from MRL and MRL/lpr mice, were carefully harvested after perfusion with phosphate buffered saline. The adipose tissues were gently blotted and individually weighed.

### Histology and immunohistochemistry

Thoracic aortas (with PVAT) and kidneys were fixed in 10% neutral formalin prior to being embedded in paraffin and sectioned into 5 μm thick serial cross sections by Augusta University Histopathology Laboratory. Tissues were stained with hematoxylin and eosin (H&E) and images captured using DP74 microscope camera (Olympus). To quantify adipocyte size and size distribution, uncompressed *tif* files were analyzed for adipocyte area by the Adiposoft plugin (v.1.16) from National Institutes of Health (NIH) ImageJ software, with each cell being individually identified by lipid inclusion (empty fields) in the tissue. Adipocytes ranging from 25-5000 μm^2^ in area were included. Three slides were prepared from each mouse and ~ 300-450 adipocytes were analyzed. Relative frequency of adipocyte area was calculated. To evaluate vascular remodeling, aortic sections were stained using Masson’s Trichrome (MT) and Verhoeff-Van Gieson (VVG). Three sections from each mouse at 300-μm intervals were analyzed. The lumen, internal elastic lamina (IEL), external elastic lamina (EEL) and adventitia were defined, and the medial area was calculated (tissue between IEL and EEL) using NIH ImageJ. Immunohistochemical (IHC) staining was performed on paraffin-embedded sections of thoracic aortas (with PVAT) following standard procedures by incubating the sections with a primary antibody against uncoupling protein 1 (UCP1, 1:800; Abcam, ab234430, MA) or CD45 (1:1000; Abcam, ab281586, MA) at 4^o^C overnight. After washing, the sections were incubated with ImmPRESS-AP Horse Anti-Rabbit IgG Polymer kit (Vector Laboratories, Burlingame, CA) and developed. To examine mast cell distribution in the PVAT, the slides were stained with 0.1% Toluidine Blue solution (PH 2.3) (Sigma; 89640-5G) for 2 minutes, followed by rinsing in distilled water for 3 exchanges and then dehydrated rapidly by applying 95% and 100% alcohol. Images were captured using DP74 (Olympus). Areas of positive staining were quantified using NIH ImageJ.

### RNA extraction and quantitative reverse transcription PCR

Total RNA was isolated from PVAT using the RNeasy Lipid Tissue Mini Kit (QIAGEN) according to the manufacturer’s instruction. Purity of total RNA was determined as 260/280 nm absorbance ratio with expected values between 1.8 to 2.2 using Biodrop Duo (Biochrom, Holliston, MA). cDNA was synthesized from 500 ng total RNA using the OneScript® Hot cDNA Synthesis Kit (abm G594, Richmond, Canada). Quantitative reverse transcription PCR (RT-PCR) was performed with BlasTaq™ 2x qPCR Master Mix (abm G892, Richmond, Canada) and the StepOne Plus™ Real-Time PCR System (Applied Biosystems, Waltham, MA) following the manufacturer’s instructions. Relative mRNA levels were determined using acidic ribosomal phosphoprotein P0 (*Arbp*) as an endogenous control gene for adipogenic/thermogenic and inflammatory markers, and *Gapdh* an endogenous control gene for immune cell markers. Sequences of the primers used for PCR amplification are listed in **Supplementary Material Table S1**.

### Statistical analysis

All data were presented as mean ± standard error of the mean (SEM). To calculate statistical significance, a nonparametric Mann-Whitney test was used after determining the distribution and variance of the data. One-way analysis of variance (ANOVA) followed by Tukey’s multiple comparisons test was used when more than two independent groups were compared. All t-tests were two-tailed, and a value of P<0.05 was considered statistically significant. All statistical analyses were conducted with Prism 9 software (Graphpad, La Jolla, CA).

## Results

### Partial lipodystrophy in MRL/lpr mice

**Figure 1:**
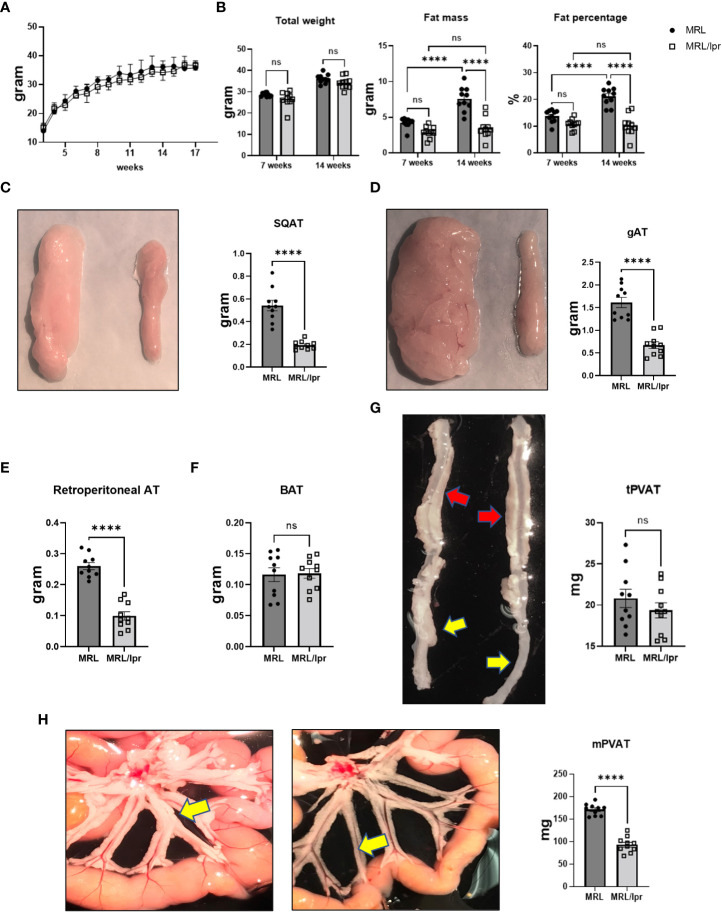
MRL/lpr mice with active disease exhibited partial lipodystrophy. **(A)** Body weights were monitored weekly. **(B)** Total weight, fat mass and fat percentage at baseline (7 weeks) and with active disease (14 weeks) measured by NMR. **(C-G)** Representative images and weights of adipose tissue depots. Subcutaneous (SQAT, **(C)**, gonadal (gAT, **(D)**, retroperitoneal **(E)**, brown (BAT, **(F)**, thoracic aortic PVAT (tPVAT, red arrows, **(G)** and abdominal aortic PVAT (aPVAT, yellow arrows, **(G)** and mesenteric arterial PVAT (mPVAT, yellow arrows, **(H)**. In all images, MRL tissues are depicted on the left and MRL/lpr on the right. n=10-15. ****p<0.0001 vs MRL (control). ns, not significant.

MRL/lpr mice recapitulate clinical manifestations and immune dysregulation observed in human lupus, including skin lesions, lymphadenopathy, splenomegaly, elevated autoantibodies such as anti-dsDNA antibodies, and renal disease at age of 14 to 20 weeks (**Supplementary Material Figure S1**). Body weight was measured weekly throughout the experiment, and body fat composition was determined using NMR spectroscopy. While both MRL/lpr and control MRL mice had similar body weights throughout the study (**Figures 1A, B**), MRL/lpr mice with active lupus displayed reduced fat mass (**Figure 1B**), characterized by decreased weights of subcutaneous, gonadal, and retroperitoneal (back of kidney) adipose tissues, as well as mesenteric PVAT (**Figures 1C–E, G, H**). There were no differences, however, in weights of brown adipose tissue or thoracic aortic PVAT between lupus and control mice (**Figures 1F, G**). Interestingly, in contrast to control mice, fat mass and percentage did not change over time in lupus mice (**Figure 1B**).

### MRL/lpr mice were hypermetabolic

**Figure 2:**
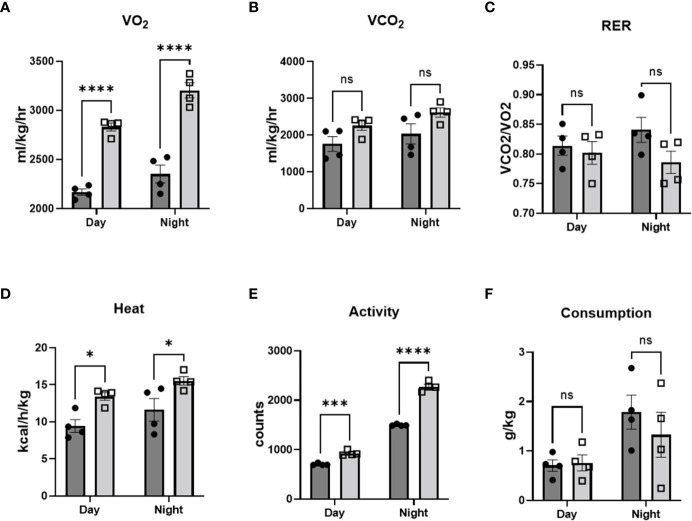
MRL/lpr mice were hypermetabolic. Comprehensive laboratory animal monitoring system (CLAMS) was performed at age of 14 weeks. VO_2_ **(A)**, CO_2_ **(B)**, heat production **(D)**, activity **(E)**, and food consumption **(F)** were measured, and respiratory exchange ratio **(C)** was calculated. n=4. *p<0.05, ***p<0.001, ****p<0.0001 vs MRL (control). ns, not significant.

In order to investigate metabolic phenotype in our lupus model, we performed indirect calorimetry test using comprehensive laboratory animal monitoring system (CLAMS). Oxygen consumption was increased in MRL/lpr mice (**Figure 2A**), while CO_2_ production (**Figure 2B**) and RER (calculated as the ratio of CO_2_ production to O_2_ consumption) (**Figure 2C**) were similar to that observed in control mice. The increased oxygen consumption in MRL/lpr mice was accompanied by increases in heat production/energy expenditure (**Figure 2D**) and locomotor activity (**Figure 2E**), while the rate of food intake (**Figure 2F**) was not significantly different between lupus and control mice. These results suggest that lupus mice are hypermetabolic, without alterations in energy substrate utilization. The increased heat generation occurring throughout the measurement period, even during times of relative inactivity (**Figure 2D**), implies that the increased energy expenditure in MRL/lpr mice is in part metabolically-driven, and not solely due to increased locomotor activity. Interestingly, despite the hypermetabolic state, MRL/lpr mice had lower core temperature compared to control mice in ambient environment (20-22°C) (**Supplementary Material Figure S2**), which may reflect insufficient insulating capacity due to loss of subcutaneous fat.

### MRL/lpr mice displayed normal blood pressure, insulin sensitivity, glucose tolerance and dyslipidemia

**Figure 3:**
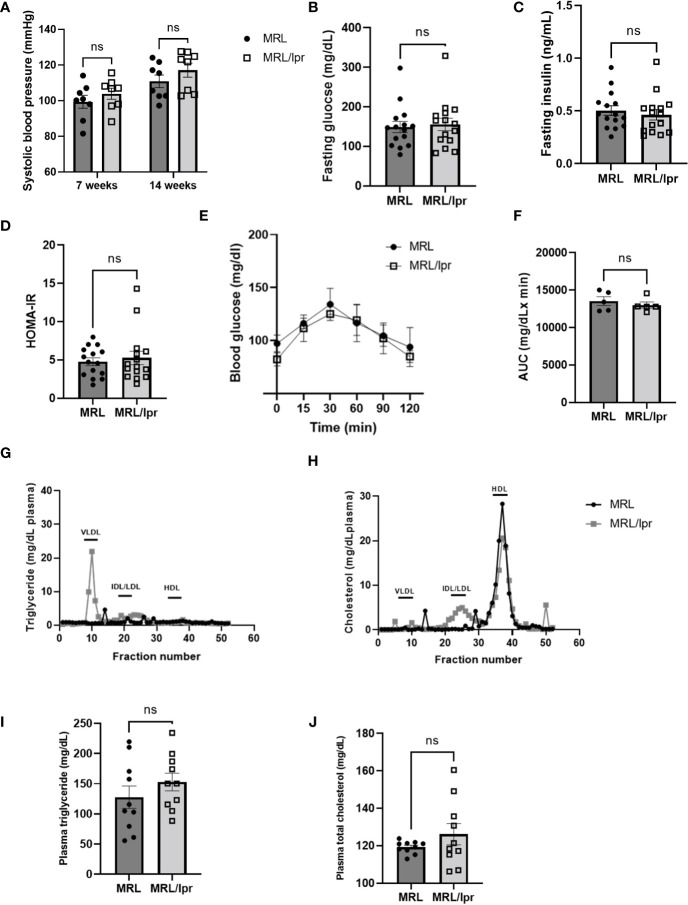
MRL/lpr mice with active disease displayed normal blood pressure and metabolic health. Systolic blood pressure **(A)** was measured using tail cuff (n=8). Fasting blood glucose **(B)** and insulin **(C)** levels were measured, and estimated insulin sensitivity as insulin resistance index (HOMA-IR) **(D)** was calculated using the University of Oxford HOMA calculator software (n=15). Glucose tolerance tests (GTT, **(E)** were performed and the area under the curve (AUC, **(F)** between MRL/lpr and MRL was compared (n=5). **(G)** and **(H)** representative plasma lipoprotein profiles measured *via* FPLC using pooled plasma (0.22 ml) from 4 MRL/lpr mice and 5 MRL mice. Plasma levels of triglyceride **(I)** and cholesterol **(J)** were also measured using biochemical assays (n=10). VLDL, very low-density lipoprotein; IDL, intermediate-density lipoprotein; LDL, low-density lipoprotein; HDL, high-density lipoprotein. ns, not significant.

Although systolic blood pressure, measured using tail cuff, was increased over time (up to 14 weeks), there was no significant difference in blood pressure between lupus and control mice (**Figure 3A**). Next, we measured fasting glucose and insulin levels and estimated insulin sensitivity using the insulin resistance index (HOMA-IR), calculated using the University of Oxford HOMA calculator software as: [fasting glucose (mmol/L) × fasting insulin (µIU/mL)] ÷ 22.5. No significant differences were detected between lupus and control mice in fasting glucose (**Figure 3B**), fasting insulin (**Figure 3C**), or HOMA-IR (**Figure 3D**). Moreover, we performed glucose tolerance tests (GTTs), which showed similar results in lupus and control mice (**FigureS 3E, F**). In addition, there were no significant differences in levels of plasma total triglycerides (**Figure 3I**) or cholesterol (**Figure 3J**) between lupus and control mice. However, MRL/lpr mice displayed higher levels of very-low-density lipoprotein (VLDL)-triglyceride [MRL/lpr (21.9 mg/dL) vs MRL (4.6 mg/dL)], but not VLDL-cholesterol, compared with control mice (**Figures 3G, H**). Moreover, MRL/lpr mice showed increased levels of intermediate-density lipoprotein (IDL)/low-density lipoprotein (LDL)-cholesterol [(MRL/lpr (5.0 mg/dL) vs MRL (0.9 mg/dL)] and reduced high-density lipoprotein (HDL)-cholesterol [(MRL/lpr (20.6 mg/dL) vs MRL (28.3 mg/dL)] (**Figure 3H**). No significant differences in lipid contents of other plasma lipoprotein species, including IDL/LDL-triglyceride, HDL-triglyceride and VLDL-cholesterol, were noted in MRL/lpr mice (**Figures 3G, H**). These findings suggest that MRL/lpr mice exhibit selective abnormalities in lipoprotein profile while maintaining normal baseline parameters of other systemic cardiovascular and metabolic health, such as blood pressure, glucose and insulin tolerance.

### Vascular endothelial function was impaired in MRL/lpr mice and exacerbated by PVAT

**Figure 4:**
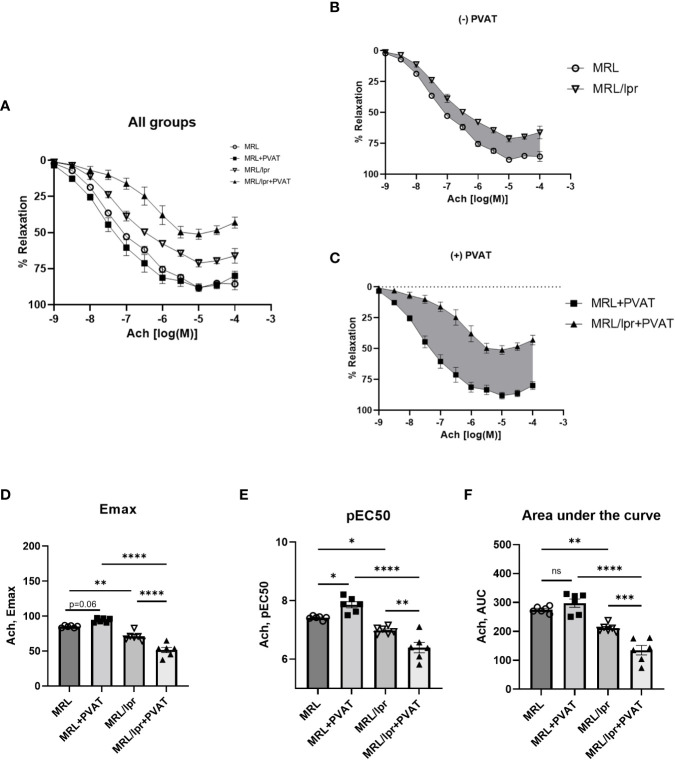
MRL/lpr mice exhibited impaired endothelium-dependent relaxation, which was exacerbated by PVAT. **(A)** Concentration-response curves to Ach in all groups. **(B)** Concentration-response curves to Ach in the absence of PVAT. **(C)** Concentration-response curves to Ach in the presence of PVAT. Gray shaded areas indicate reductions in Ach-induced relaxation in MRL/lpr mice. **(D)** Maximum responses (Emax) to Ach from all concentration-response curves. **(E)** Negative logarithm of EC50 (pEC50) for all concentration-response curves. **(F)** Area under the curve (AUC) for all concentration-response curves. *p < 0.05, **p < 0.01, ***p < 0.001, ****p < 0.0001. Ach, acetylcholine; PVAT, perivascular adipose tissue. ns, not significant.

To evaluate vascular function, thoracic aortas were isolated with or without PVAT from MRL/lpr and control mice at age of 14 weeks, and isometric tension was recorded using a wire myograph system. Consistent with previous reports, MRL/lpr mice exhibited impaired endothelium-dependent vasorelaxation to acetylcholine (Ach) in thoracic aortas (**Figures 4B, D-F**). To investigate the role of PVAT in regulating endothelial function, we tested aortas with or without associated PVAT. Interestingly, the endothelial dysfunction in MRL/lpr mice was further augmented by the presence of PVAT (**Figure 4**) as evidenced by decreased Emax (D), pEC50 (E), and AUC (F), while the presence of PVAT tended to enhance endothelium-dependent relaxation in control mice (**Figures 4A-F**). In contrast, endothelium-independent vasorelaxation to sodium nitroprusside (SNP) was unaffected by presence of PVAT in MRL/lpr or control mice (**Supplementary Material Figure S3**). We further evaluated endothelial function in a different lupus-prone mouse model (NZBWF1/J mice). As was observed in MRL/lpr mice, endothelial dysfunction was exacerbated by the presence of PVAT in lupus NZB/W mice. Note that the NZB/W mouse study was conducted at 40 weeks of age, at which time PVAT appeared to lose its anti-contractile function in the control mice (**Supplementary Material Figure S4**). These findings suggest that the detrimental effects of PVAT on endothelial function in MRL/lpr mice were not related to Fas-Fasl signaling associated with the MRL/lpr background. Collectively, these findings suggest that PVAT exacerbates endothelial dysfunction in the setting of active lupus.

### PVAT “whitening” and vascular remodeling in MRL/lpr mice

**Figure 5:**
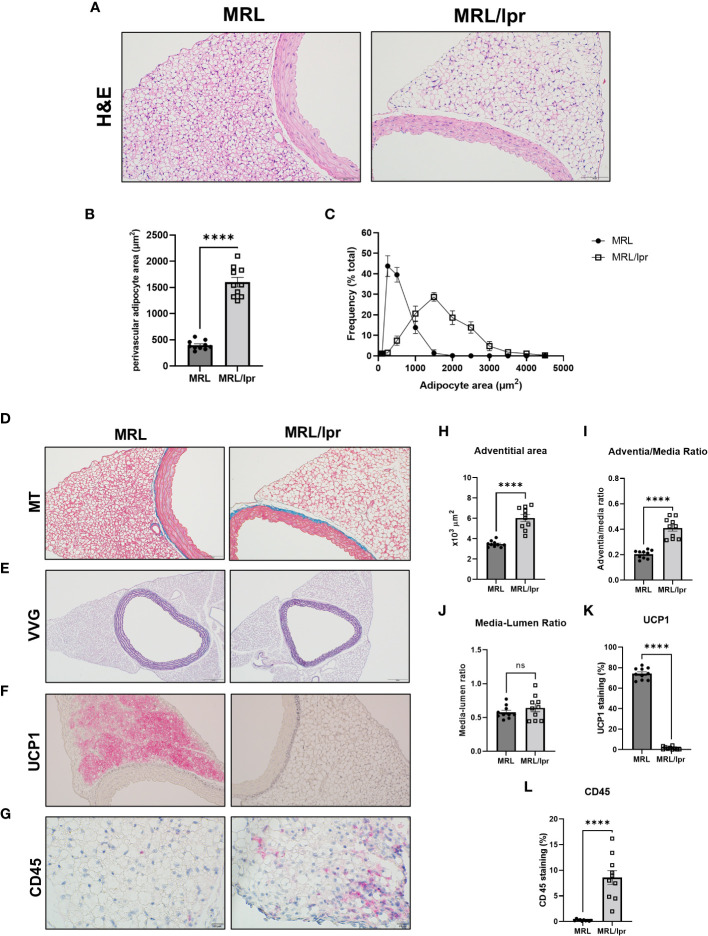
PVAT exhibited whitening and vascular remodeling in MRL/lpr mice. **(A)** Hematoxylin and eosin (H&E), scale bar=100 μm. **(B)** Quantitative analysis of average adipocyte size (area per adipocyte, μm^2^); **(C)** the relative distribution of adipocytes calculated as frequency of adipocyte area (%) over total area. **(D)** Masson Trichrome (MT) staining for collagen, scale bar=100 μm; **(E)** Verhoeff-Van Gieson (VVG) staining for elastin, scale bar=200 μm; **(F)** UCP1 staining for beige adipocytes, scale bar=100 μm; **(G)** CD45 staining for leukocytes, scale bar=20 μm; and **(H-L)** quantification of adventitial areas, adventitia-media ratio, media-lumen ratio, percentage of positive UCP1 and CD45 areas. n=10. ****p < 0.0001 vs MRL (control). ns, not significant.

PVAT is brown-like in healthy mice, and whitening of PVAT is associated with abnormal vascular homeostasis. To characterize the morphological features of PVAT from lupus mice, histology and immunohistochemical analysis was performed. We analyzed adipocyte size distribution in PVAT. Adipocytes were significantly hypertrophic in the PVAT of MRL/lpr mice compared to control mice (1601 ± 91.03 μm^2^ vs 396.1 ± 27.14 um^2^, respectively, **Figures 5A, B**). In addition, while control mice exhibited a beige adipocyte dominant pattern, characterized by the majority of adipocytes being < 250 μm^2^, lupus mice exhibited a flattening and a shift to the right of the adipocyte size distribution curves, indicating an increased proportion of large adipocytes (**Figure 5C**). Furthermore, collagen deposition (**Figures 5D, H**, Masson Trichrome staining – blue color) was increased in PVAT from lupus mice, which was most evident at the adventitial border zone. Additionally, adventitial hyperplasia (**Figures 5E, I**) was also observed, whereas no difference was seen in elastin staining or media-lumen ratio (**Figures 5E, J**). Notably, as compared to control mice, expression of UCP1, a beige adipocyte marker, (**Figures 5F, K**), was reduced, while leukocyte recruitment as examined by CD45 immunostaining (**Figures 5G, L**) was increased, in PVAT from lupus mice. While mast cells play an important role in the pathogenesis of SLE, there was no difference in mast cell recruitment to PVAT of lupus mice compared with control mice, as determined by toluidine blue staining (**Supplementary material Figure S5A**) or mast cell-specific gene expression (CD117, **Supplementary material Figure S5B**). Furthermore, plasma levels of mast cell specific protease-6 (Mcpt6) were similar in lupus mice and control mice (**Supplementary Material Figure S5D**), further suggesting that mast cells are most likely not responsible for the phenotypic changes in PVAT observed in lupus mice.

### PVAT from MRL/lpr mice exhibited increased expression of pro-inflammatory genes, and decreased expression of adipogenic and beige genes

**Figure 6:**
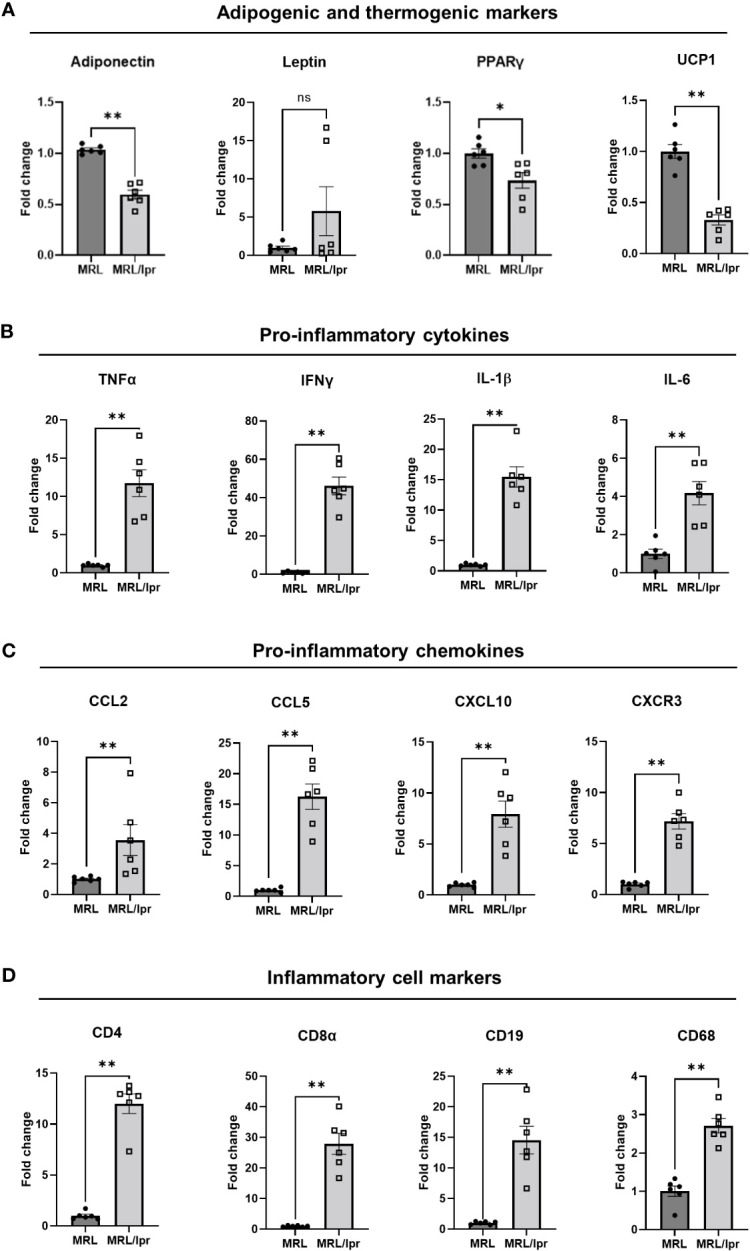
PVAT from MRL/lpr mice exhibited decreased adipogenic and thermogenic marker genes **(A)**, and increased proinflammatory genes including cytokines **(B)**, chemokines **(C)**, and inflammatory marker **(D)** genes. n=6. *p<0.05, **p < 0.01, vs MRL (control). ns, not significant.

Next, we quantified expression of inflammatory, adipogenic, and metabolic genes in PVAT from lupus mice. Interestingly, adiponectin expression (**Figure 6A**) was significantly reduced in PVAT from lupus mice, concordant with decreased plasma levels of adiponectin (**Supplementary Material Figure S4A**). While mRNA expression of pro-inflammatory leptin in PVAT was similar in lupus mice versus control mice (**Figure 6A**), plasma leptin levels were decreased in lupus mice (**Supplementary Material Figure S4A**). Adipogenic (PPARγ) and thermogenic (UCP1) marker gene expression was significantly decreased in PVAT from lupus mice (**Figure 6A**). Importantly, expression of pro-inflammatory cytokines (IL-1β, IL-6, TNFα and IFNγ) (**Figure 6B**) was increased in PVAT from lupus mice, concomitant with elevated plasma levels of TNFα, IFNγ and IL-6 (**Supplementary Material Figure S3B**). In addition, multiple chemokines, including CCL2, CCL5, and CXCL10, were expressed at higher levels in PVAT and plasma from lupus mice (**Figure 6C**, **Supplementary Figure S3C**). The expression of markers of T cells (CD4, CD8α), B cells (CD19) and macrophages (CD68) was elevated in PVAT from lupus mice (**Figure 6D**). These findings suggest that inflammation originating in PVAT could induce whitening, impair thermogenic and metabolic activities, and promote the local release of factors that perturb endothelial function in lupus mice.

## Discussion

Inflammation promotes endothelial dysfunction and formation of intimal lesions in atherosclerosis. Increasing evidence suggests that the outer layers of the arterial wall, including the adventitia and PVAT, may promote vascular inflammation and endothelial dysfunction through an “outside-in” mechanism. Here, we used lupus-prone mice to examine the role of PVAT in regulating vascular function in lupus, a disease associated with chronic systemic inflammation and increased risk of atherosclerotic CVD (30). We report for the first time that thoracic aortic PVAT of lupus mice exhibits: (1) dysfunctional features, as evidenced by “whitening” and hypertrophy of perivascular adipocytes, and immune cell infiltration; (2) reduced expression of adipogenic and beige adipocyte genes, including adiponectin, PPARγ, and UCP1; and (3) increased expression of pro-inflammatory cytokines and chemokines. This dysfunctional and inflamed PVAT in lupus mice was associated with impaired endothelium-dependent relaxation and adventitial remodeling. Collectively, these data suggest that dysfunctional PVAT contributes to CVD risk in the context of lupus.

Generalized loss of adipose tissues in subcutaneous and visceral compartments, a condition known as lipodystrophy, is associated with insulin resistance, dyslipidemia, endothelial dysfunction, and predisposition to atherosclerosis (31, 32). In our study, female MRL/lpr lupus mice with active disease were partially lipodystrophic and dyslipidemic but exhibited normal blood pressure, insulin sensitivity, and glucose tolerance. Nevertheless, the lupus mice exhibited endothelial dysfunction of thoracic aorta, which was aggravated by the presence of PVAT. Taken together, these findings suggest that lipodystrophy *per se* is not the sole cause of endothelial dysfunction in lupus mice and imply a distinct pathogenic role for PVAT in this process. We did not systematically investigate the biology or function of other adipose depots in our mice, nor did we examine the impact of PVAT on resistance microvasculature responsible for blood pressure control. However, Choi et al. demonstrated that male MRL/lpr mice, which likewise harbor a Fas mutation but do not typically develop lupus at early age, exhibited reduced subcutaneous, visceral, and brown adipose tissue mass, decreased adipocyte size in subcutaneous adipose tissues, and enhanced glucose tolerance. Moreover, these mice exhibited increased white (epididymal and inguinal subcutaneous) adipose tissue UCP1 expression and browning after a cold challenge and were resistant to high-fat diet induced obesity (33). This latter finding is consistent with the hypermetabolic state noted in our female MRL/lpr mice housed at ambient temperature. Furthermore, Wueest et al. reported that Fas expression was increased in adipocytes isolated from insulin-resistant mice and in adipose tissues of obese and diabetic patients, and that deletion of Fas in adipocytes decreased adipose tissue inflammation, hepatic steatosis, and insulin resistance induced by a high-fat diet (34). In order to exclude an effect of Fas-FasL signaling associated with the MRL/lpr background, we evaluated endothelial function in another lupus-prone mouse model (NZB/W mice) in the absence or presence of PVAT. Consistent with MRL/lpr mice, lupus-prone NZB mice exhibited exacerbation of endothelial dysfunction in the presence of PVAT. Taken together, these findings suggest that PVAT itself may uniquely drive endothelial dysfunction in lupus.

Like adipose depots in other anatomic locations, PVAT contains both preadipocytes and mature adipocytes with distinct gene expression and functional profiles (19, 35). The PVAT surrounding thoracic aorta in healthy rodents and humans was reported to be enriched in beige adipocytes (36). Similarly to brown adipocytes, beige adipocytes exhibit increased mitochondrial biogenesis, multilocular lipid droplets, and elevated expression of UCP1 (37). Thermogenic activity of healthy perivascular adipocytes can also increase vascular lipid clearance. Smooth muscle cell selective PPARγ (SMPG) knockout mice, which lack PVAT, exhibited impaired thermogenic activity and endothelial dysfunction. Moreover, cold exposure inhibited atherosclerosis in mice with intact PVAT, but not in SMPG knockout mice, suggesting a potentially protective role of thermogenic PVAT in atherosclerosis (38). Interestingly, mice with active lupus exhibit loss of thermogenic activity in PVAT, as evidenced by reduced expression of UCP1 and phenotypic “whitening,” characterized by reduced multilocular lipid droplets and increased adipocyte size. These findings suggest that active lupus is associated with conversion of perivascular adipocytes from beige to white, in conjunction with loss of healthy PVAT’s atheroprotective functions.

Under homeostatic conditions, PVAT inflammation is low, and perivascular adipocytes primarily secrete relaxing factors and anti-inflammatory adipokines, such as adiponectin, which inhibits vascular inflammation and plaque formation (39) and improves endothelial function through endothelial NO synthase phosphorylation (40). Moreover, adiponectin may reduce vascular smooth muscle cell proliferation and migration (41, 42). By contrast, in atherosclerosis and following vascular injury, PVAT is heavily infiltrated by immune cells (21, 43, 44) and predominantly produces contracting factors such as norepinephrine and reactive oxygen species, as well as pro-inflammatory cytokines and chemokines such as IL-1β, IL-6, TNFα, CCL2 (19, 20, 45, 46). This imbalance in relaxing and contractile factors, and anti- and pro-inflammatory factors is strongly linked to vascular inflammation and disease (39). The PVAT in lupus mice exhibited increased infiltration of immune cells and expression of pro-inflammatory cytokines (e.g., IL-1β, IL-6, TNFα, IFNγ), along with decreased expression of adiponectin, which likely promotes endothelial dysfunction and vascular wall remodeling. The subtypes of immune cells, and the mechanisms responsible for their recruitment to PVAT, remain to be determined. Also, the mechanism of dyslipidemia, the impact of high fat diet on PVAT, and the subsequent influence on endothelial dysfunction and atherosclerotic lesion formation in lupus mice will require further investigations. Finally, our data suggest that the increased collagen content and adventitial remodeling detected in lupus mice might impose an anatomical barrier to outside-in signaling from PVAT to the vascular wall. Whether and how this might impact lupus-related vascular disease is unclear.

Although MRL/lpr mice have been widely accepted and used as a mouse model of lupus disease, they do not perfectly mimic the clinical parameters of lupus patients. Our MRL/lpr mice were lean and exhibited reduced PVAT mass, whereas many patients with lupus have obesity, metabolic syndrome, and/or increased volume of thoracic PVAT. Medications (such as steroids), high caloric “western” diets, and sedentary behavior likely contribute to these differences between mice and humans with lupus. Additionally, humans typically live in comfortable, temperature-controlled environments, whereas the mice used in this study were kept at 20-22°C, well below their thermoneutral zone (28-32°C). This causes murine brown/beige adipocytes to utilize energy for heat production, thus diminishing the size of fat mass depots. Further studies are required to determine how changes in diet, activity and environmental temperature modulate PVAT function in lupus mice, and the subsequent impact on CVD.

In conclusion, our findings suggest that active lupus is associated with dysfunctional, inflamed PVAT, which may lead to impaired endothelial-dependent vasorelaxation and aberrant vascular remodeling. These findings may have important clinical implications for lupus-related CVD.

## Data availability statement

The original contributions presented in the study are included in the article/**Supplementary Material**. Further inquiries can be directed to the corresponding author.

## Ethics statement

The animal study was reviewed and approved by Institutional Animal Care & Use Committee of Augusta University.

## Author contributions

HS, HWK and NLW developed the conception and design of the study, HS, BG, DK, TCK, MO, DYH and JM performed experiments and collected data, HW, EJB, DS, XL, AG, RL, LC, BHA, HWK and NLW analyzed, interpreted and discussed data, HS, HWK and NLW wrote the manuscript.
