# Supplementary material for: Perivascular adipose tissue promotes vascular dysfunction in murine lupus
Source: Front Immunol. 2023 Mar 16;14:1095034. doi: 10.3389/fimmu.2023.1095034 (PMC10062185; doi:10.3389/fimmu.2023.1095034)
Supplement: Supplementary file 1 [file Presentation_1.pptx]

## Slide 1
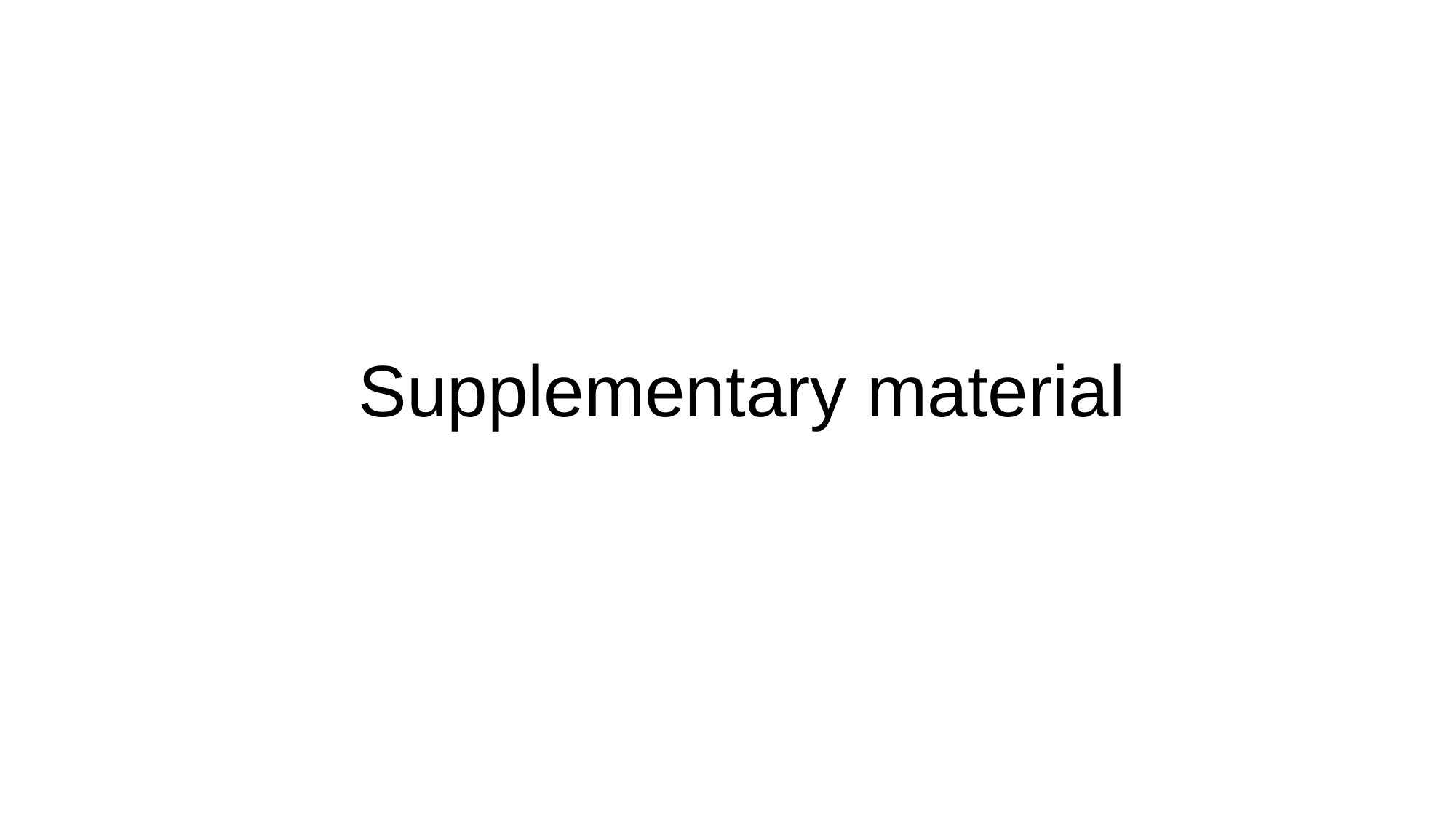

# Supplementary material

## Slide 2
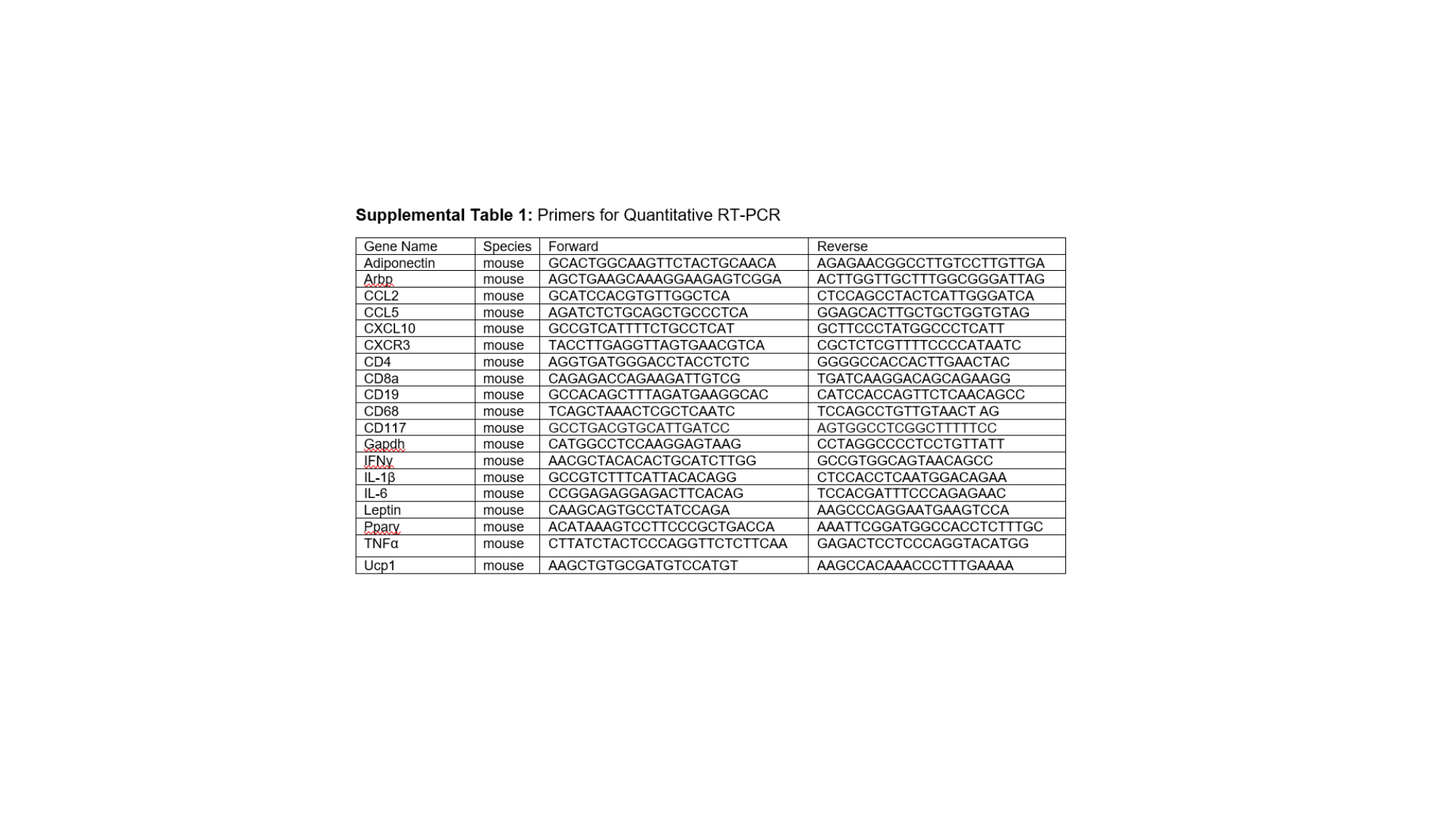

## Slide 3
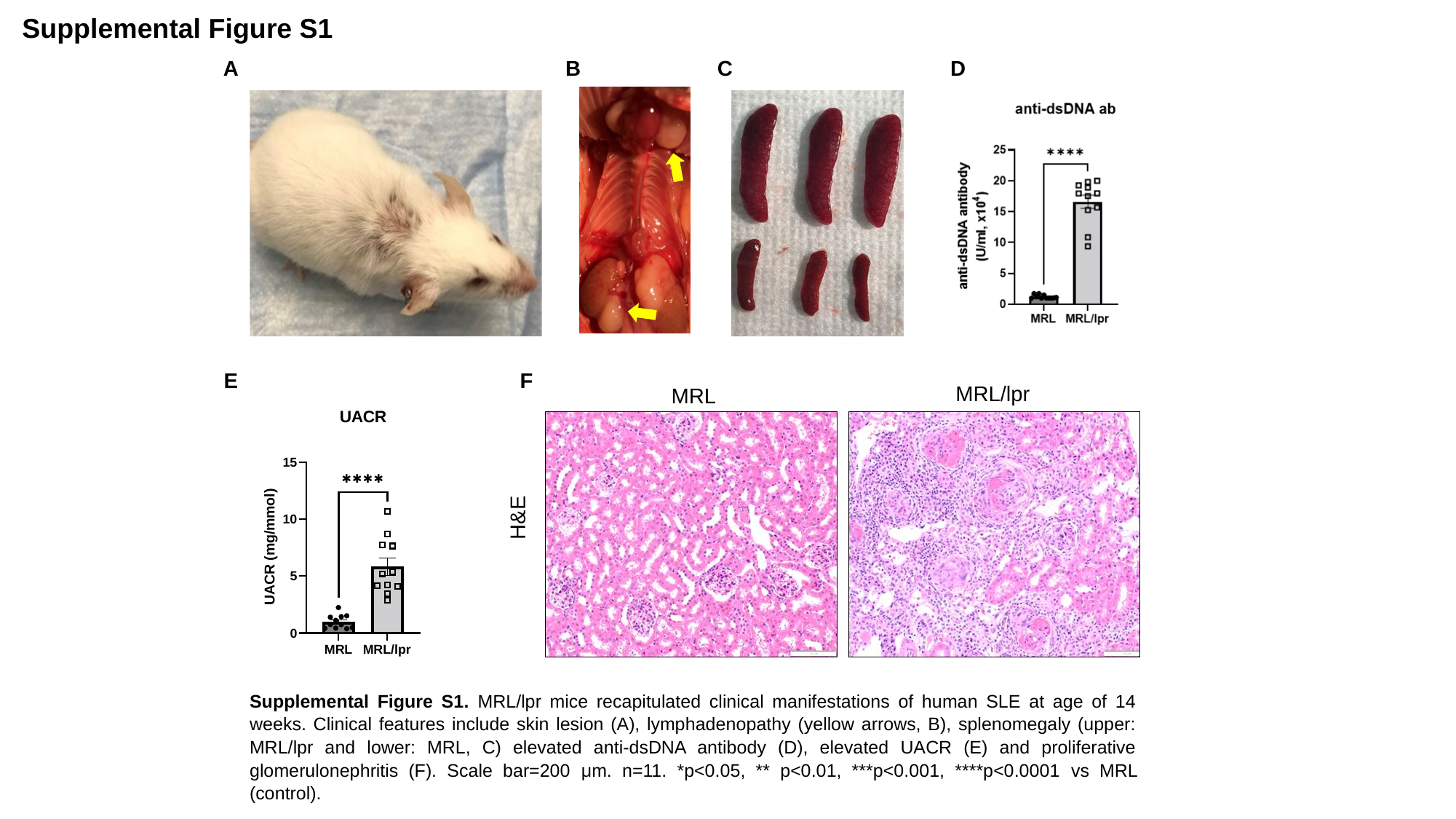

Supplemental Figure S1
D
C
A
B
E
F
MRL/lpr
MRL
H&E
Supplemental Figure S1. MRL/lpr mice recapitulated clinical manifestations of human SLE at age of 14 weeks. Clinical features include skin lesion (A), lymphadenopathy (yellow arrows, B), splenomegaly (upper: MRL/lpr and lower: MRL, C) elevated anti-dsDNA antibody (D), elevated UACR (E) and proliferative glomerulonephritis (F). Scale bar=200 μm. n=11. *p<0.05, ** p<0.01, ***p<0.001, ****p<0.0001 vs MRL (control).

## Slide 4
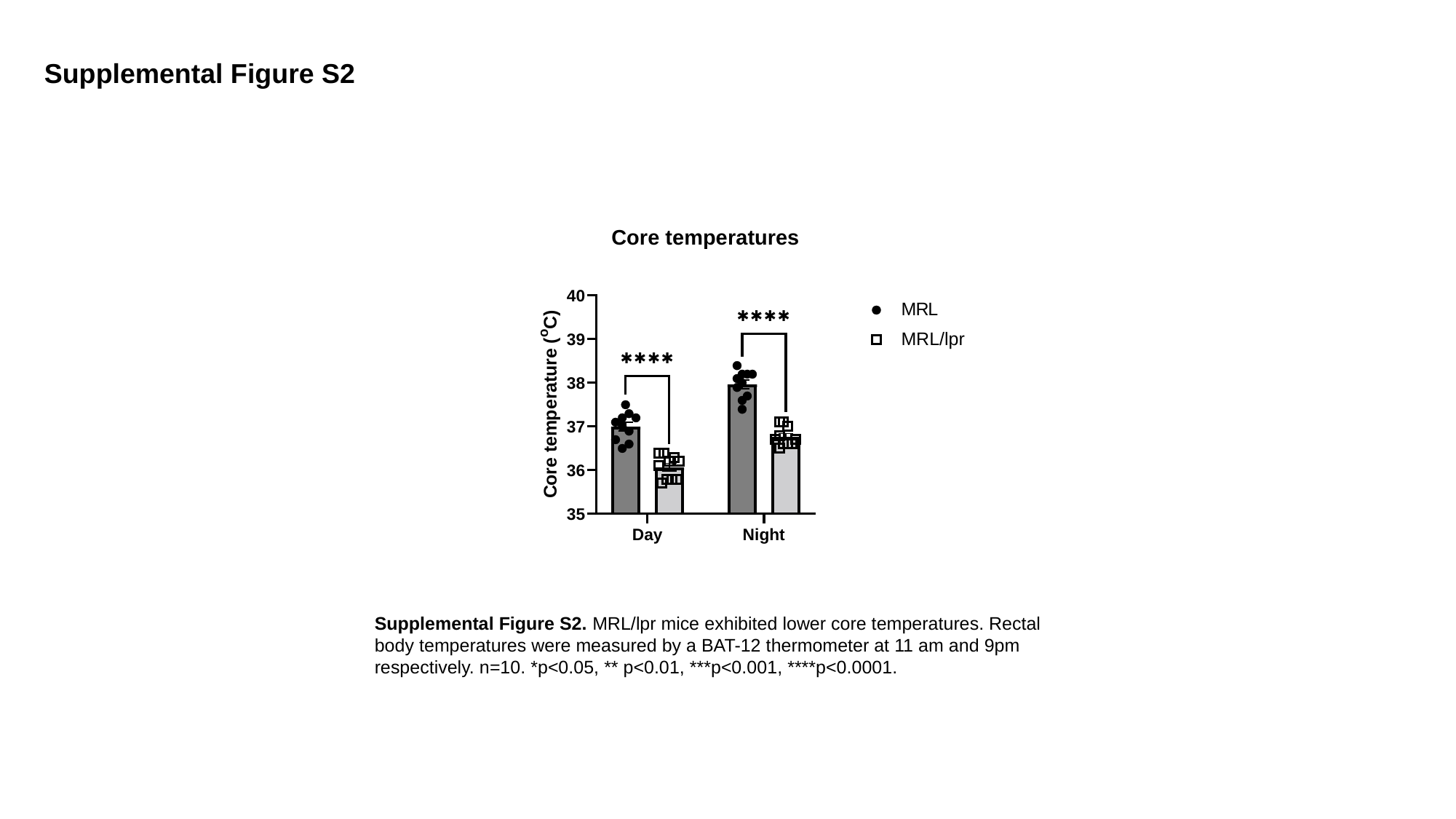

Supplemental Figure S2
Supplemental Figure S2. MRL/lpr mice exhibited lower core temperatures. Rectal body temperatures were measured by a BAT-12 thermometer at 11 am and 9pm respectively. n=10. *p<0.05, ** p<0.01, ***p<0.001, ****p<0.0001.

## Slide 5
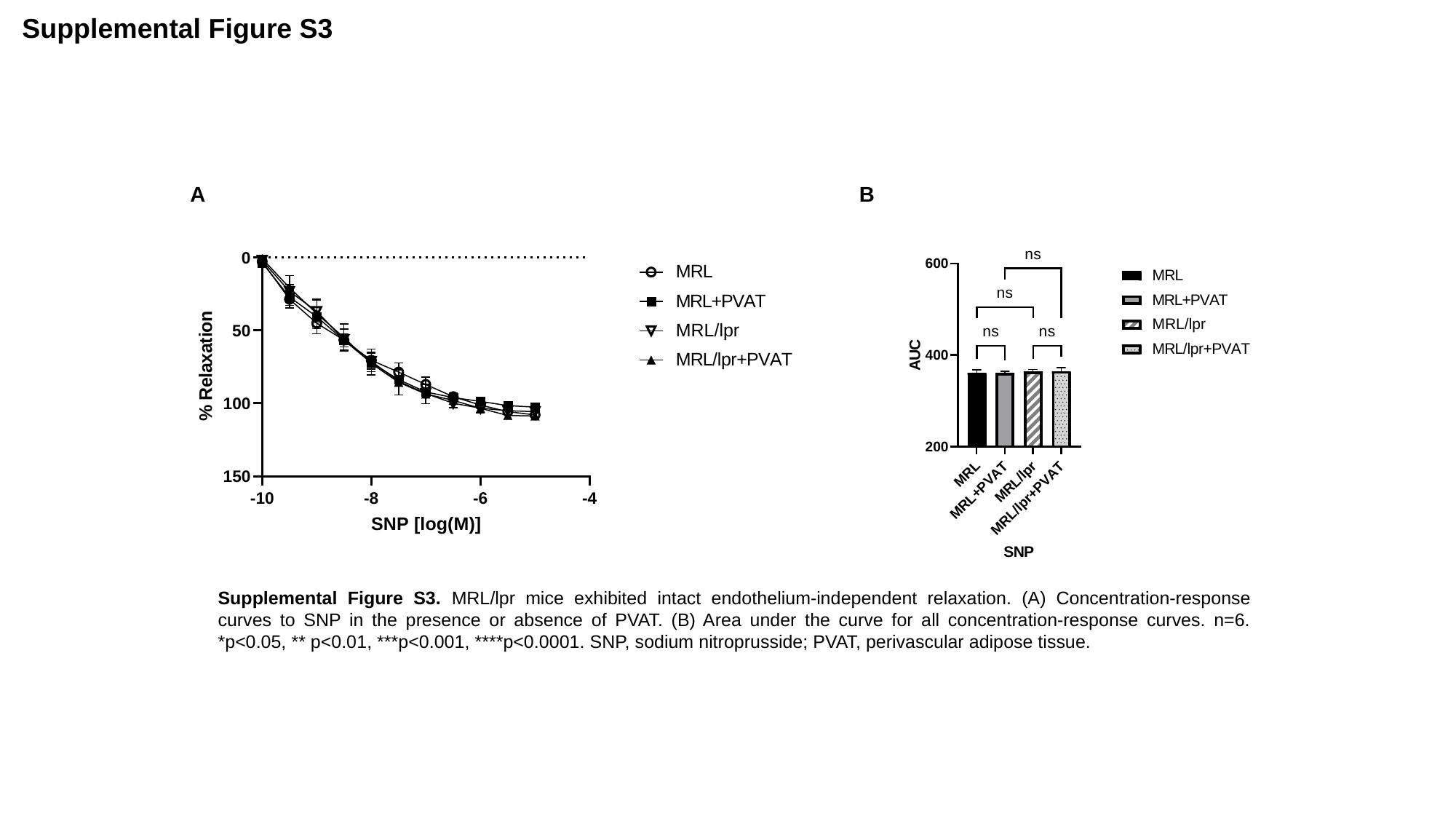

Supplemental Figure S3
B
A
Supplemental Figure S3. MRL/lpr mice exhibited intact endothelium-independent relaxation. (A) Concentration-response curves to SNP in the presence or absence of PVAT. (B) Area under the curve for all concentration-response curves. n=6. *p<0.05, ** p<0.01, ***p<0.001, ****p<0.0001. SNP, sodium nitroprusside; PVAT, perivascular adipose tissue.

## Slide 6
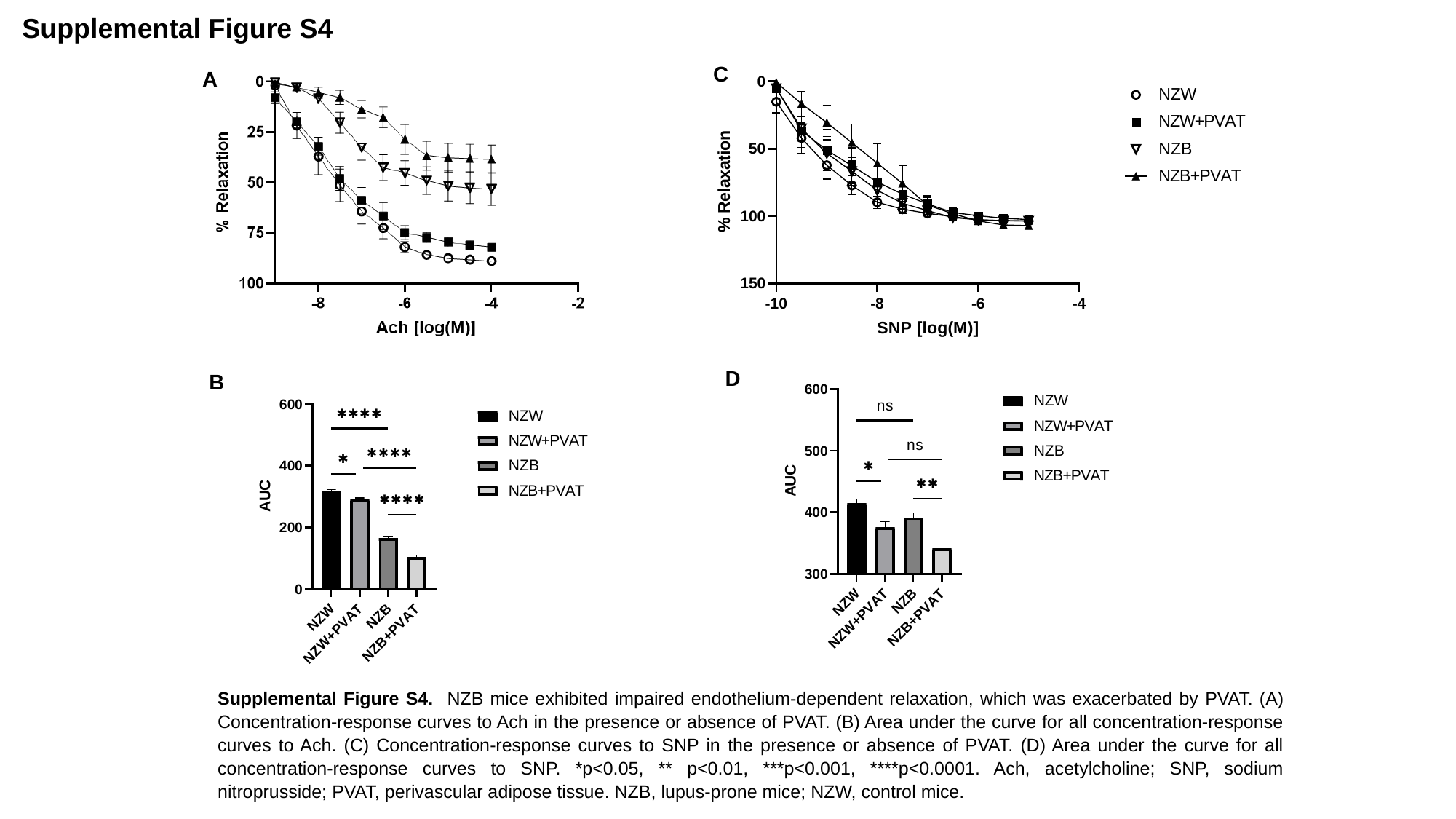

Supplemental Figure S4
C
A
D
B
Supplemental Figure S4. NZB mice exhibited impaired endothelium-dependent relaxation, which was exacerbated by PVAT. (A) Concentration-response curves to Ach in the presence or absence of PVAT. (B) Area under the curve for all concentration-response curves to Ach. (C) Concentration-response curves to SNP in the presence or absence of PVAT. (D) Area under the curve for all concentration-response curves to SNP. *p<0.05, ** p<0.01, ***p<0.001, ****p<0.0001. Ach, acetylcholine; SNP, sodium nitroprusside; PVAT, perivascular adipose tissue. NZB, lupus-prone mice; NZW, control mice.

## Slide 7
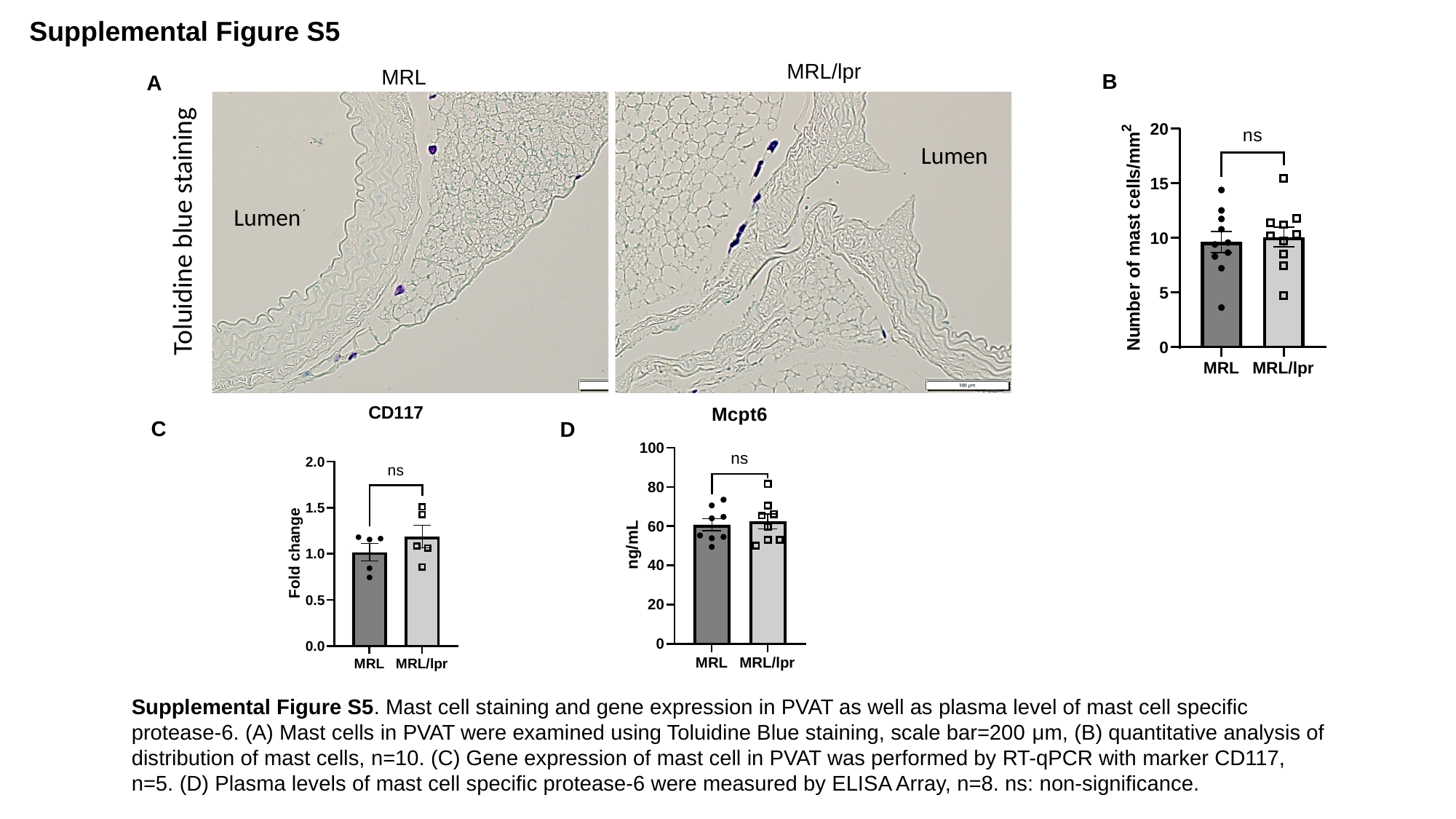

Supplemental Figure S5
MRL/lpr
MRL
B
A
Toluidine blue staining
Lumen
Lumen
C
D
Supplemental Figure S5. Mast cell staining and gene expression in PVAT as well as plasma level of mast cell specific protease-6. (A) Mast cells in PVAT were examined using Toluidine Blue staining, scale bar=200 μm, (B) quantitative analysis of distribution of mast cells, n=10. (C) Gene expression of mast cell in PVAT was performed by RT-qPCR with marker CD117, n=5. (D) Plasma levels of mast cell specific protease-6 were measured by ELISA Array, n=8. ns: non-significance.

## Slide 8
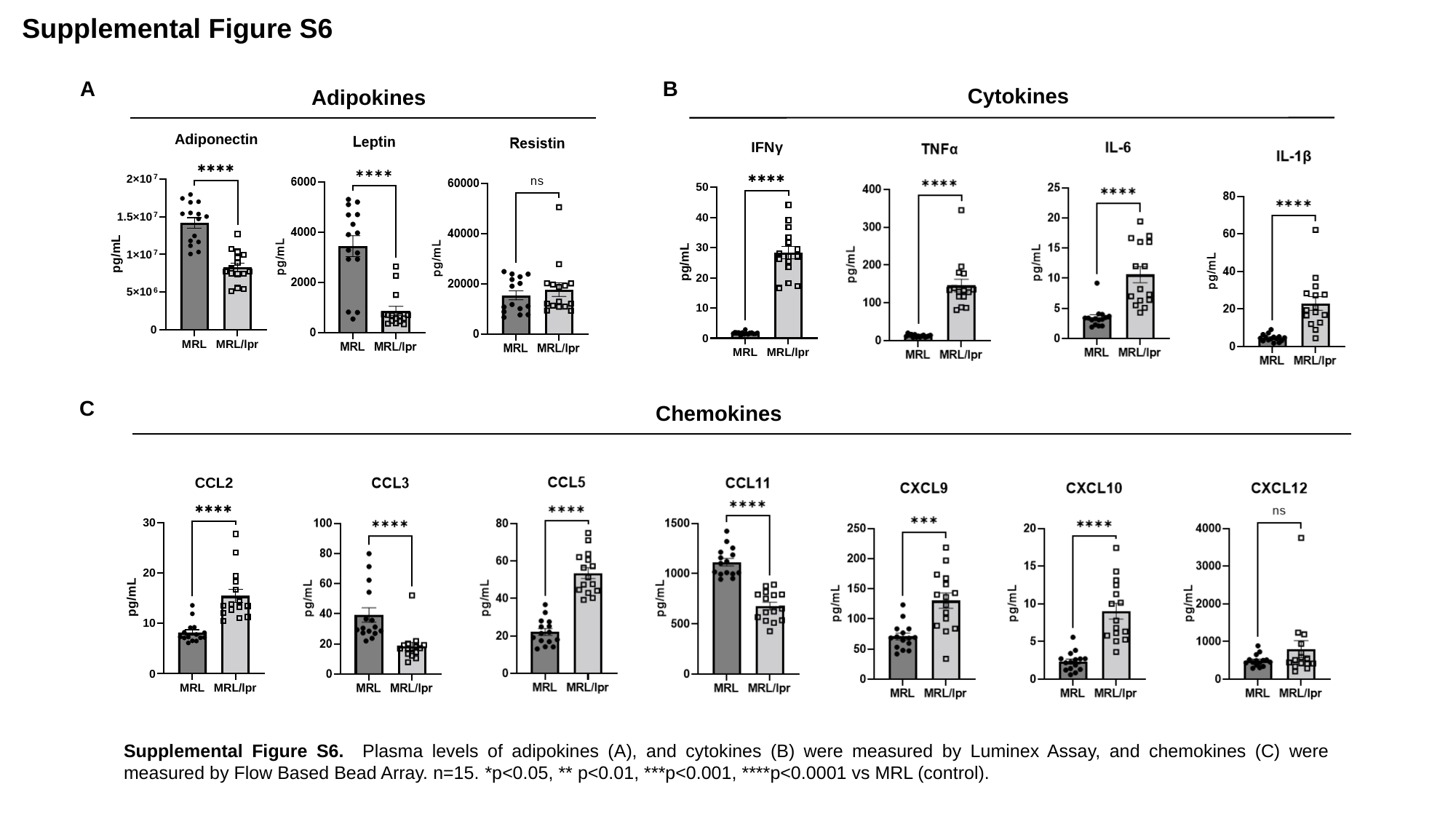

Supplemental Figure S6
A
B
Cytokines
Adipokines
C
Chemokines
Supplemental Figure S6. Plasma levels of adipokines (A), and cytokines (B) were measured by Luminex Assay, and chemokines (C) were measured by Flow Based Bead Array. n=15. *p<0.05, ** p<0.01, ***p<0.001, ****p<0.0001 vs MRL (control).
